# Hesperidin abrogates bisphenol A endocrine disruption through binding with fibroblast growth factor 21 (FGF-21), α-amylase and α-glucosidase: an in silico molecular study

**DOI:** 10.1186/s43141-022-00370-z

**Published:** 2022-06-01

**Authors:** P. M. Aja, J. N. Awoke, P. C. Agu, A. E. Adegboyega, E. M. Ezeh, I. O. Igwenyi, O. U. Orji, O. G. Ani, B. A. Ale, U. A. Ibiam

**Affiliations:** 1 Department of Biochemistry, Faculty of Science, Ebonyi State University, Abakaliki, Nigeria; 2 Department of Biochemistry, Faculty of Medical Sciences, University of Jos/Jaris Computational Biology Centre, Jos, Nigeria; 3 Department of Chemical Engineering, Nnamdi Azikiwe University, Awka, Nigeria; 4 Nutrition and Exercise Physiology, University of Missouri, Columbia, United States of America; 5 Department of Biochemistry, University of Nigeria Nsukka, Nsukka, Nigeria; 6 Interdisciplinary Biomedical Research Centre, School of Science and Technology, Nottingham Trent University, Nottingham, UK

**Keywords:** Fibroblast growth factor-21, α-amylase, α-glucosidase, Bisphenol A, Hesperidin, Endocrine disruption chemicals

## Abstract

**Background:**

Fibroblast growth factor 21 (FGF-21), alpha-amylase, and alpha-glucosidase are key proteins implicated in metabolic dysregulations. Bisphenol A (BPA) is an environmental toxicant known to cause endocrine dysregulations. Hesperidin from citrus is an emerging flavonoid for metabolic diseases management. Through computational approach, we investigated the potentials of hesperidin in abrogating BPA interference in metabolism. The 3D crystal structure of the proteins (FGF-21, α-amylase, and α-glucosidase) and the ligands (BPA and hesperidin) were retrieved from the PDB and PubChem database respectively. Using Autodock plugin Pyrx, molecular docking of the ligands and individual proteins were performed to ascertain their binding affinities and their potentials to compete for the same binding site. Validation of the docking study was considered as the ability of the ligands to bind at the same site of each proteins. The docking poses were visualized using UCSF Chimera and Discovery Studio 2020, respectively to reveal each of the protein-ligands interactions within the binding pockets. Using SwissAdme and AdmeSar servers, we further investigated hesperidin’s ADMET profile. Hesperidin used was purchased commercially.

**Results:**

Hesperidin and BPA competitively bound to the same site on each protein. Interestingly, hesperidin had greater binding affinities (Kcal/mol) − 5.80, − 9.60, and − 9.60 than BPA (Kcal/mol) − 4.40, − 7.20, − 7.10 for FGF-21, α-amylase, and α-glucosidase respectively. Visualizations of the binding poses showed that hesperidin interacted with stronger bonds than BPA within the proteins’ pockets. Although hesperidin violated Lipinski rule of five, this however can be optimized through structural modifications.

**Conclusions:**

Hesperidin may be an emerging natural product with promising therapeutic potentials against metabolic and endocrine derangement.

## Background

The prevalence of metabolic disorders is on the increase globally due to several factors including environmental toxicants such as heavy metals and several industrial products that could modulate and disrupt insulin secretion and signalling leading to dysregulations in glucose metabolism [1–3]. Previous studies have shown that multiple complex interplays between insulin secretions, signalling, and actions are responsible for efficient glucose metabolism in the body [4–6]. Hence, dysregulation of these pathways especially by endocrine-disrupting chemicals (EDC) at the genomic or proteomic levels may lead to dysfunction in the signaling molecules of glucose metabolism and consequently result in several metabolic syndromes such as obesity and diabetes [7–9]. Diabetes, for instance, typically results from a combination of peripheral insulin resistance and defects in pancreatic β-cell functions, which could be modulated by the deleterious activities of these EDC on entrance into the physiological system. Being an emerging area of research in biomedical science, the mechanisms of metabolic disruptions by various identified EDCs are poorly known; hence developing therapeutic agents to modulate their interference remains a huge task for researchers in this area.

Bisphenol A (BPA), 2,2-bis(4-Hydroxyphenyl) propane has been used in packaging materials by the food and beverage industries since the 1960s. It is one of the major structural components found in polycarbonate beverage bottles, which are commonly used by food industries. It is also used for the manufacture of epoxy and polycarbonate resins used in food containers, such as child feeding bottles and plastic bottles, or as epoxy resins coating food and beverage-containing metallic cans [10, 11]. BPA is a component in the metal can coatings, which protect food substances from directly contacting metal surfaces of the food container. Recent studies have revealed that small amounts of BPA are transferred to stored food and water from the polymer containers or from the epoxy resins lining the metallic cans, especially when exposed to high temperatures (as during sterilization cycles) [11]. Additionally, human exposure to BPA occurs through the consumption of contaminated food and drinking water, which leads to several adverse effects in the body [12, 13]. BPA has since become one of the most controversial endocrine disruptor chemicals that interferes directly with the regulation of glucose metabolism both at the genomic and proteomic levels respectively [14–16]. It has also been implicated by several studies in the pathogenesis of diabetes and other metabolic diseases [17–19]. Moreover, how BPA interferes with glucose metabolism leading to endocrine disruption in the human body remains elusive and requires more insight and mechanistic understanding.

Fibroblast growth factor 21 (FGF-21) has recently gained evident attention as one of the key endocrine hormones involved in glucose metabolism [20]. In obese diabetic rodents [21, 22] and rhesus monkeys [23, 24], studies have shown that injection of FGF-21 can result in a drastic decrease in fasting glucose, insulin, glucagon, and triglycerides. Endogenous FGF-21 functions in various physiological conditions as a stress-responsive hormone protecting the cell against distinct metabolic or environmental stress [25]. Pancreatic α-amylase and α- glucosidase are key digestive enzymes found in the epithelial mucosa of the small intestine and are involved in carbohydrate metabolism in the physiological milieu. Hence, inhibition of these enzymes can significantly reduce the post-prandial increase in blood glucose level making their therapeutic modulation essential in diabetes management [26, 27]. Hesperidin is a plant flavonoid from citrus fruits with an emerging therapeutic potential against several disease conditions such as diabetes and other metabolic syndromes [28–31]. We demonstrated most recently in our lab that hesperidin could markedly increase insulin level in cadmium-induced pancreatitis in rats [32], thus opening a new horizon for the use of hesperidin in therapeutically modulating metabolic dysregulations caused by several endocrine disruptor chemicals (EDC) that find their way into the body system. In this present study, using in silico molecular docking techniques, we investigated whether one of the molecular mechanisms of BPA endocrine disruption is through its binding with FGF-21, α-amylase, and α-glucosidase and if hesperidin could interfere with this binding, thus ameliorating metabolic dysregulations elicited by BPA.

## Methods

### Retrieval of the 3D crystal structure of target proteins

**Fig. 1:**
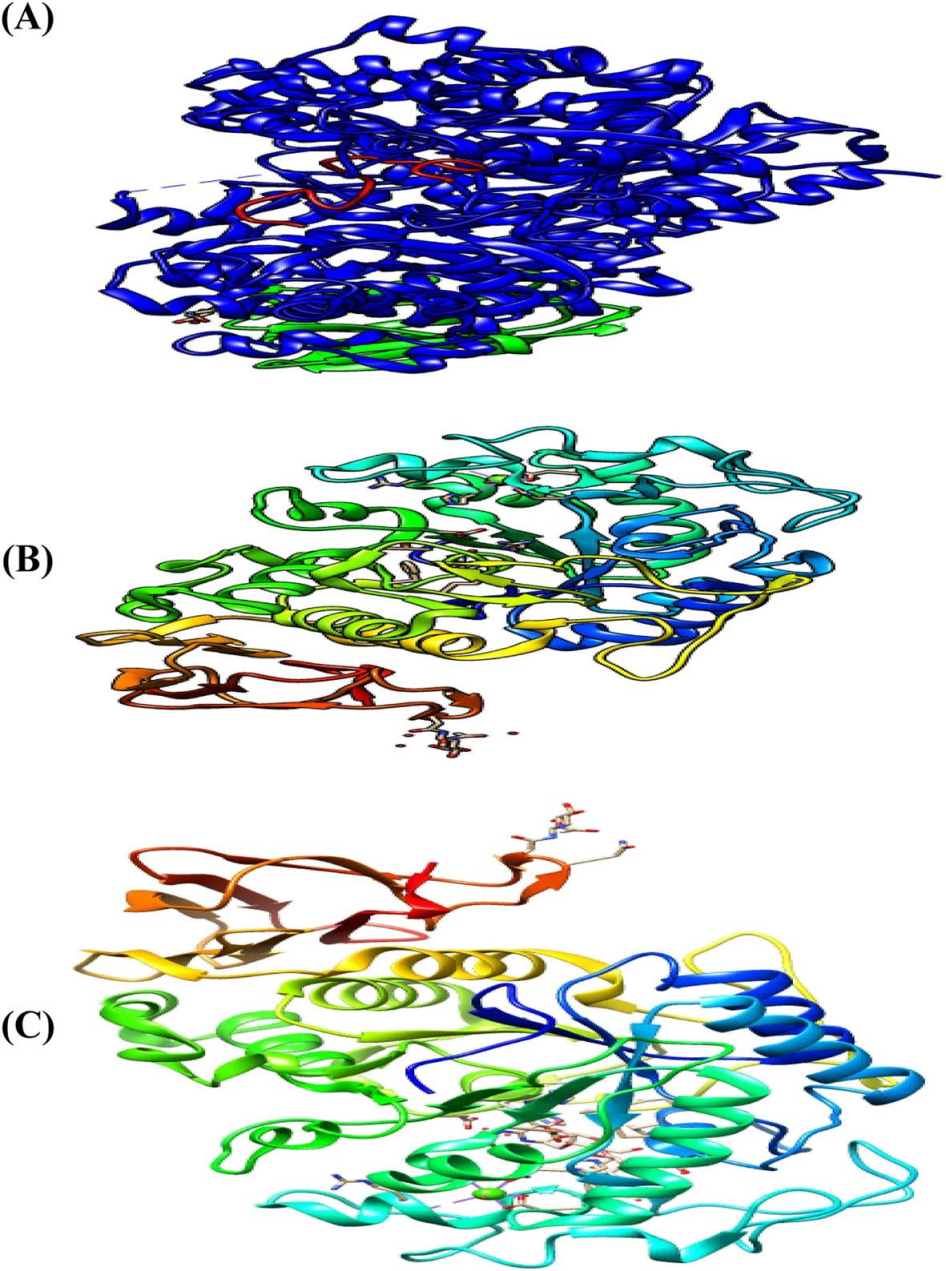
The 3D crystal structure of target proteins displayed UCSF Chimera. **(A)**. FGF-21. **(B)**. Pancreatic α-amylase. **(C)**. Pancreatic α-glucosidase

The 3D crystal structures of the proteins FGF-21 (PDB: 5VAQ), pancreatic α-amylase (PDB ID: 2QMK) pancreatic α-glucosidase (PDB ID: 1U33) were fetched by IDs from the Protein Data Bank (http://www.rcsb.org) into UCSF Chimera changed to publication quality and saved as an image (Fig. 1).

### Retrieval of the 3D structure of the ligands

**Fig. 2:**
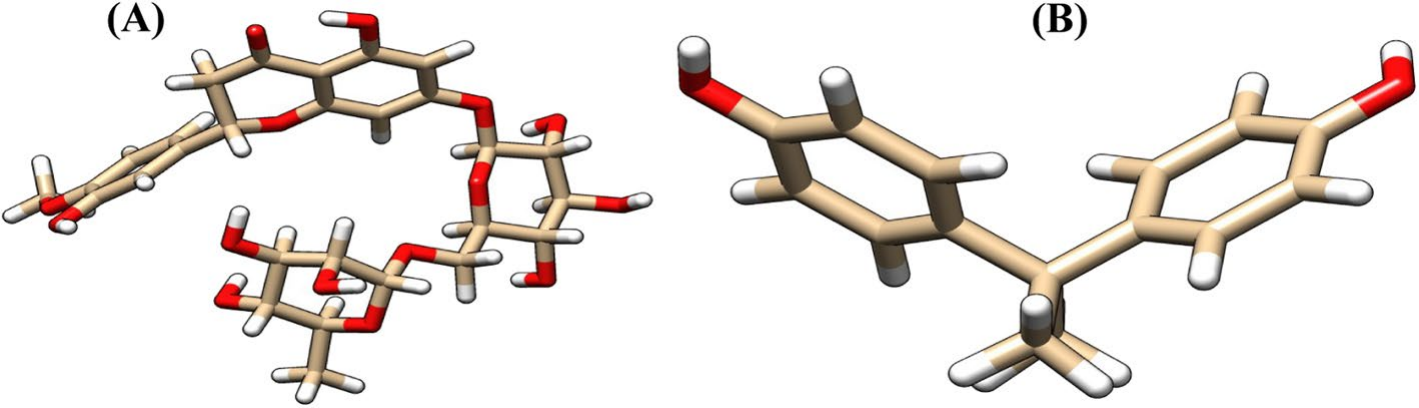
Three-dimensional structures of **(A)** hesperidin and **(B)** bisphenol A

The ligands used for the study are bisphenol A (PubChem CID: 6623) and hesperidin (PubChem CID: 10621), respectively. Their 3D structures were fetched by IDs from PubChem (www.pubchem.org) into UCSF Chimera and saved in a structure data file (SDF) and image (see Fig. 2) format, respectively.

### Preparation of the target proteins

**Fig. 3:**
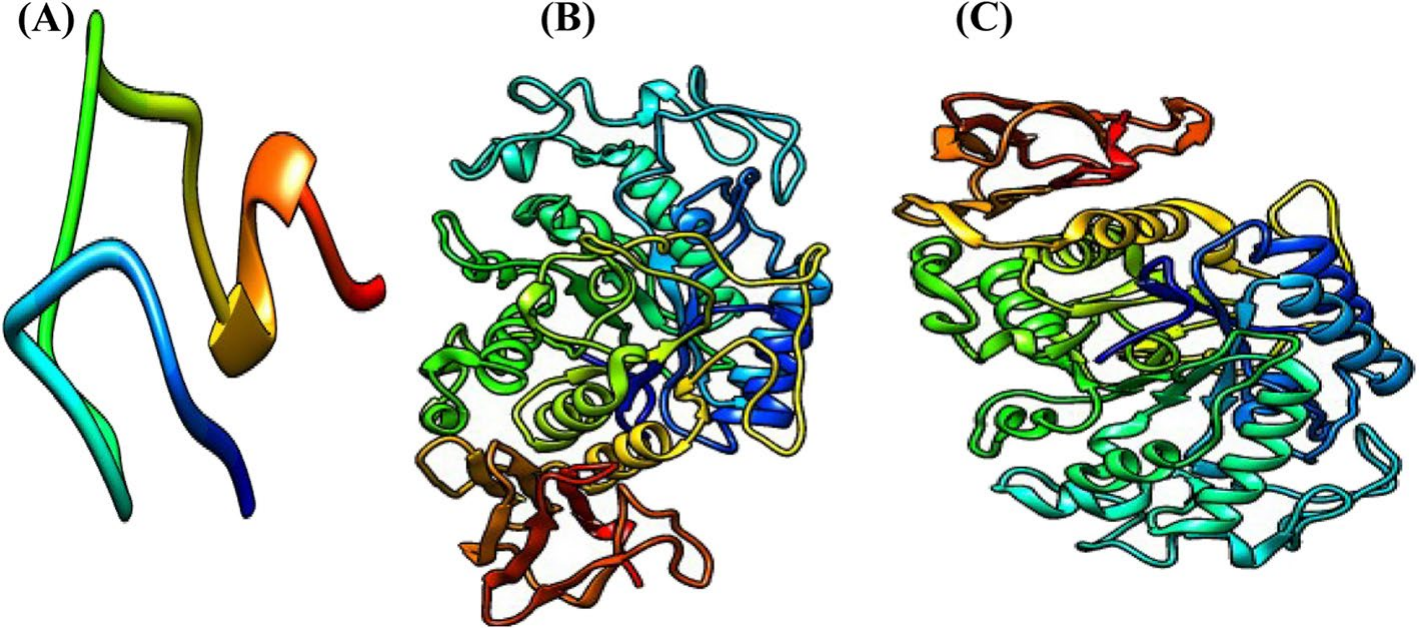
Three-dimensional crystal structure of prepared proteins. **(A)**. FGF-21. **(B)**. Pancreatic α-amylase. **(C)**. Pancreatic α-glucosidase

The target proteins were prepared and saved separately. The 3D crystal structure of each of the proteins was fetched into UCSF Chimera by their PDB ID vis-à-vis 5VAQ, 2QMK, and 1U33 respectively. The FGF-21 was in a complex with Beta-klotho and complex chains (Fig. 3A, B) were selected and deleted so that only a single-chain (Fig. 3C) which is the FGF-21 was left displayed while pancreatic α-amylase and pancreatic α-glucosidase were single chains (see Fig. 3). For each protein, structure editing was carried out by minimizing the structure under the default specifications of the steepest descent slope 100; size 0.02 Ả; gradient steps:10; conjugate 0.02; update intervals 10. After preparations, the proteins were saved in PDB format for use in molecular docking.

### Molecular docking

The molecular docking studies were carried out using Autodock Vina plugged in Pyrx. The 3D structure of the ligands (hesperidin and bisphenol A) previously retrieved from PubChem (www.pubchem.org) were individually imported into the Pyx in Chemical Table-SDF format. The ligands were minimized in the default with the addition of hydrogen and charge Gastiger, then, converted to PDBQT format. In turn, the prepared proteins as shown in Fig. 3 were loaded into the Pyrx. Using the Autodock, the proteins were made macromolecules in PDBQT format. For each turn, the program was run using a searching grid extended over target proteins with box dimension 24.26 × 31.65 × 48.11 and *x*, *y*, *z* coordinates 47.76, 32.05, and 40.10 (5VAQ); dimension 72.39 × 84.39 × 67.64 and *x*, *y*, *z* coordinates 8.02, − 29.05, 18.99 (2QMK), and 70.69 × 82.91 × 57.87 and *x*, *y*, *z* coordinates 10.09, 24.12, 48.53 (1U33) respectively [33] with exhaustiveness 8 while other parameters were set at default. The ligands were docked into the crystal structure of the proteins in turns so that the highest-scoring pose was selected for each of the ligands and the best docking poses are predicted to be the most stable conformation of each ligand for binding to proteins [34]. Selection of best pose is particularly at the binding site where the majority (or all) of the bisphenol A binds in the eighth models and if hesperidin binds with the highest docking scores in that particular site. Further, the best poses were visualized using the Discovery Studio Visualizer 2020 [35] to ascertain the amino acids in the binding sites and predict the types of bonds with which the ligands bind to the target proteins.

### ADMET predictions of hesperidin

ADMET (absorption, distribution, metabolism, excretion, and toxicity) analysis, which constitutes the pharmacokinetics of a drug-like molecule [36] was carried out on hesperidin. Here, web servers use structure-activity relationship (similarity search) to compare and predict the ADMET properties of drug candidates or environmental chemicals with properties of known compounds in their database. In this work, prediction and significant descriptors of drug-likeness such as mutagenicity, toxicological dosage level, and pharmacologically relevant properties of the hesperidin were predicted using Swissadme (http://www.swissadme.ch) and admetSAR (lmmd.ecust.edu.cn:8000) webservers.

## Results

### Docking scores and binding energy analysis

#### The best poses of the ligands with the proteins

##### Ligands’ pose with FGF-21

**Fig. 4:**
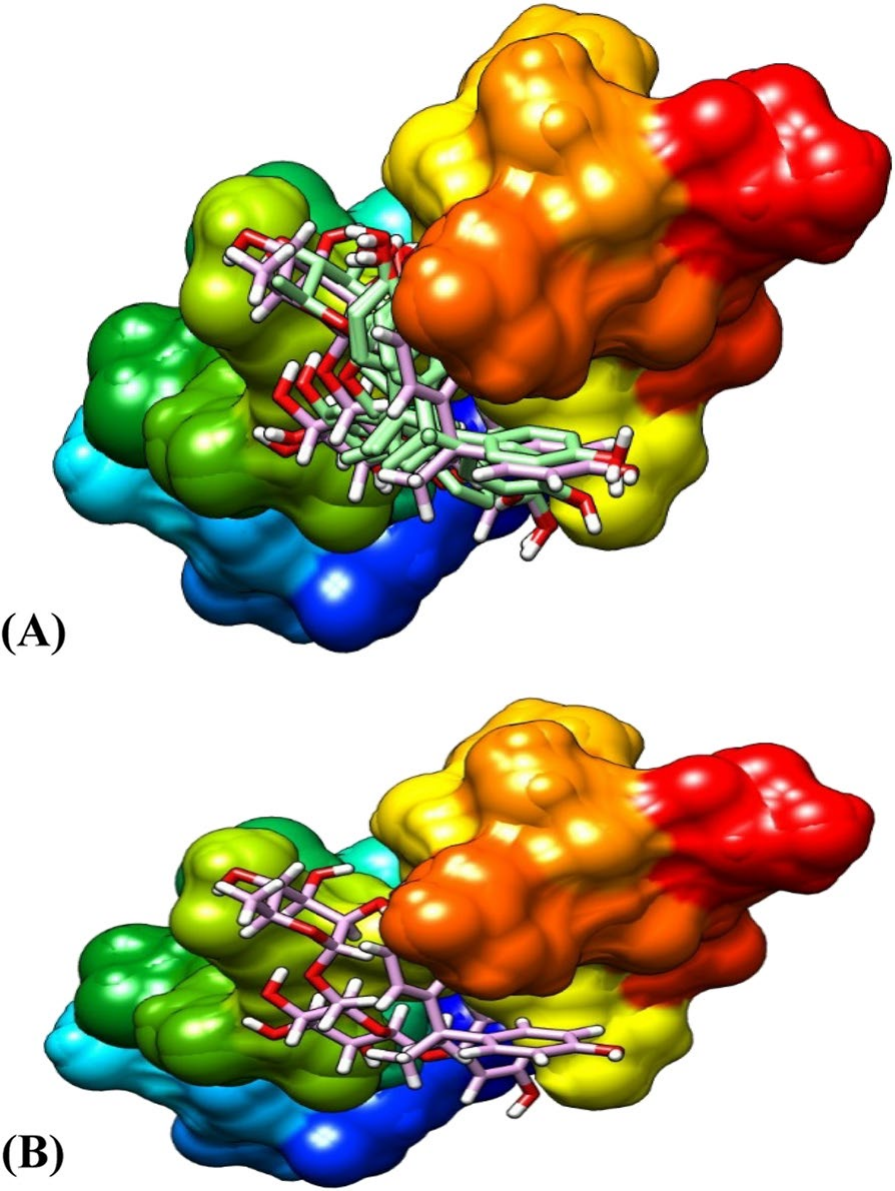
The binding pose of the ligands at the same binding site of FGF-21. **(A)**. The best model of hesperidin in complex with 8 models of bisphenol A. **(B)** The binding pose of the best conformation of the Ligands and the protein

Figure 4 shows the two best conformations (Fig. 4A, B) of the binding poses and interactions between FGF-21, hesperidin, and BPA. Figure 4A represents the best model of hesperidin in complex with all the different models of bisphenol A while Fig. 4B is the binding pose of the best conformation/models of both ligands and the protein. All the binding conformations consistently revealed that both ligands bind at the same binding pocket on the protein with hesperidin having a stronger binding affinity than all the models of BPA at the same site, which enhanced its potential of dislodging BPA from its binding pocket on the protein molecule. Hesperidin, therefore, has the potential to favourably compete for the same binding site as BPA and perhaps may inhibit the binding of BPA on its binding pocket on the protein three-dimensional conformations.

##### Ligands’ pose with α-amylase

**Fig. 5:**
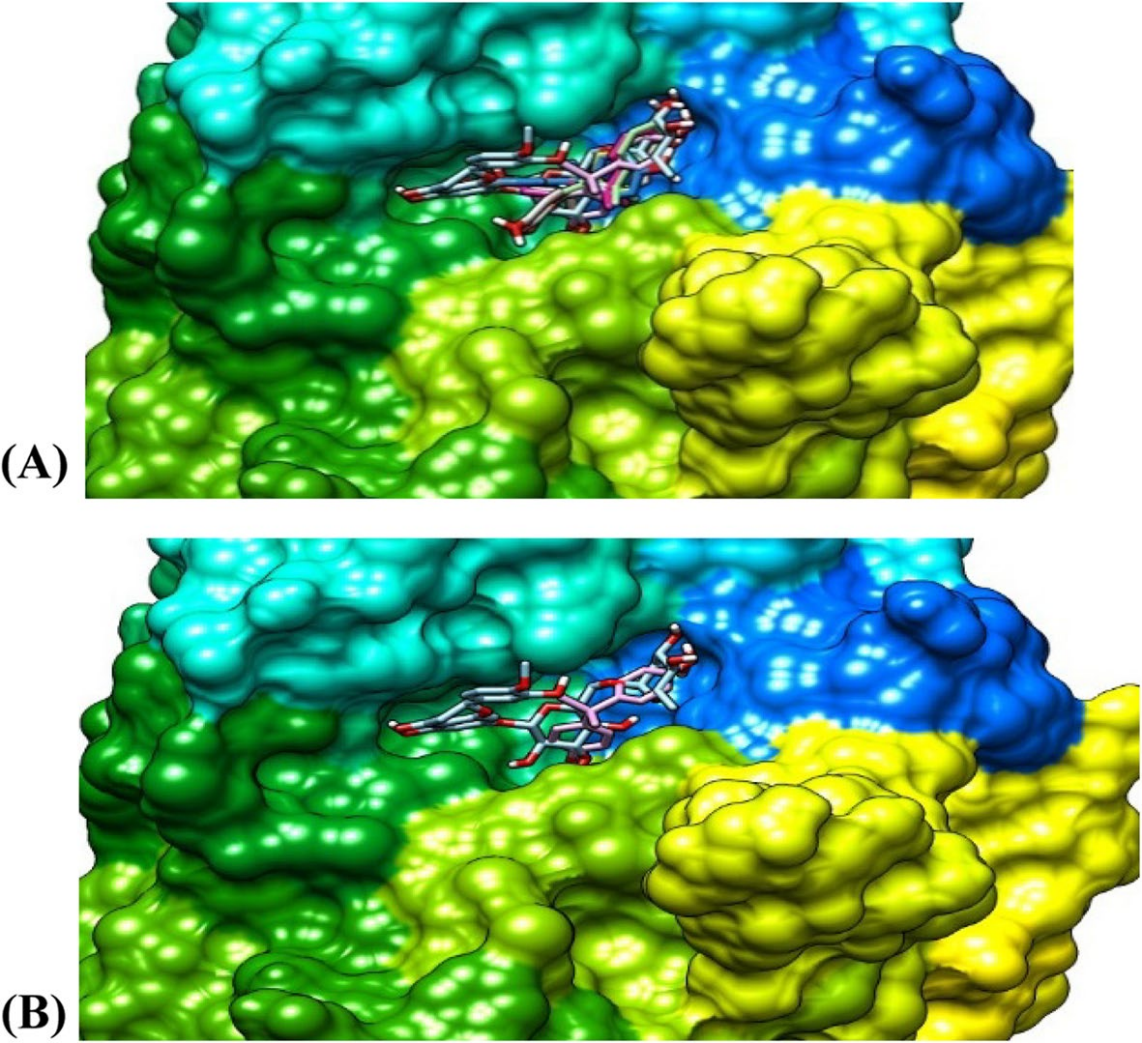
The binding pose of the ligands at the same binding site of α-amylase. **(A)**. The best model of hesperidin in complex with 7 models of bisphenol A. **(B)** The binding pose of the best conformation/models of the ligands and the protein

Figure 5 shows two best conformations (Fig. 5A, B) of the binding poses and interactions between α-amylase, hesperidin, and BPA. Figure 4A represents the best model of hesperidin in complex with seven different models of bisphenol A while Fig. 4B is the binding pose of the best conformation/models of both ligands and the protein. All the binding conformations consistently revealed that both ligands bind at the same binding pocket on the enzyme with hesperidin having a stronger binding affinity than all the models of BPA at the same site, which enhanced its potential of dislodging BPA from its binding pocket on the enzyme. Hesperidin, therefore, has the potential to compete for the same binding site as BPA and perhaps may prevent the binding of BPA on its binding pocket on the α-amylase three-dimensional enzyme conformations.

##### Ligands’ pose with α-glucosidase

**Fig. 6:**
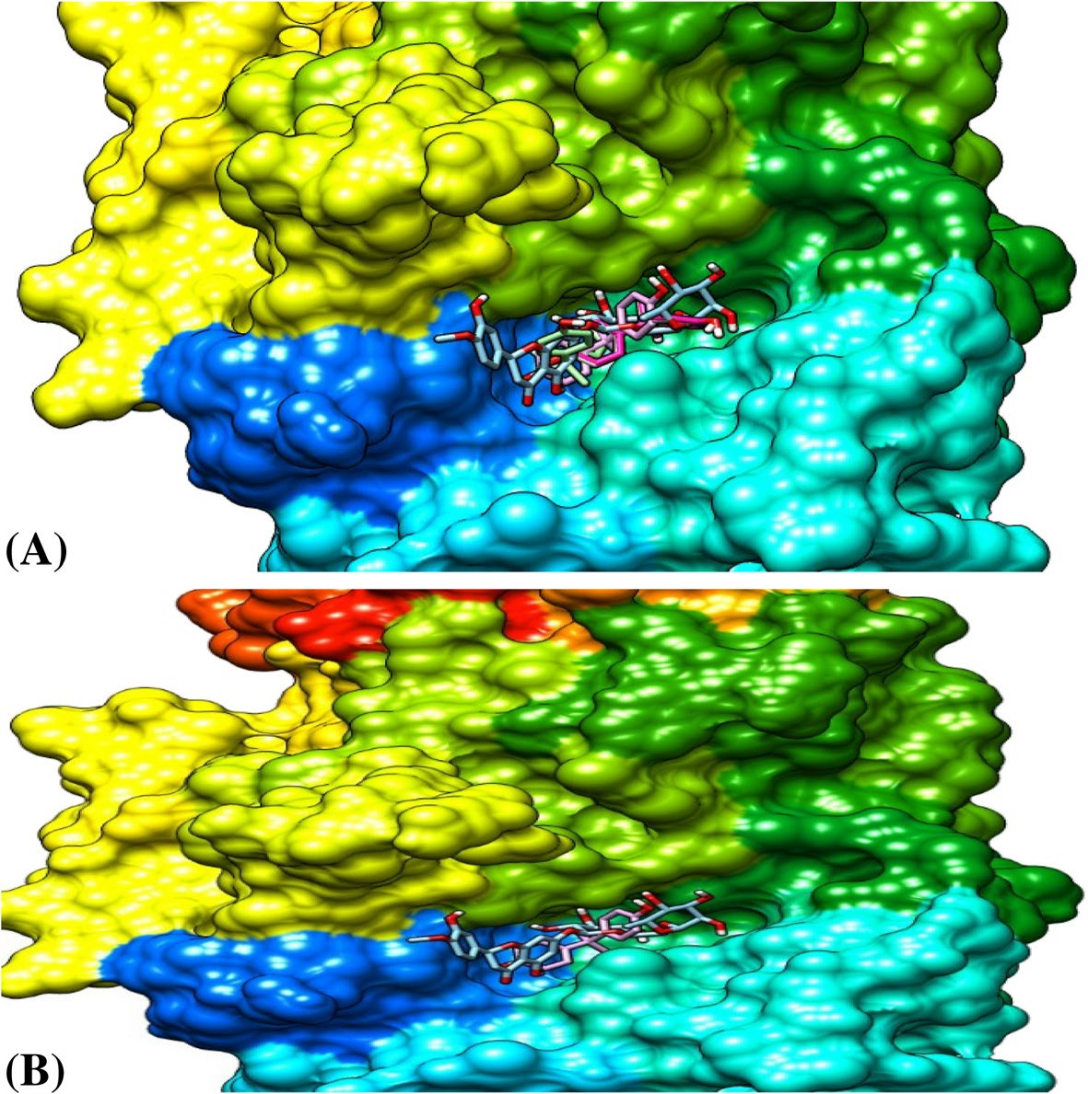
The binding pose of the ligands at the same binding site of α-glucosidase. **(A)**. The best model of hesperidin and 6 models of bisphenol A. **(B)**. The binding pose of the best conformation of the ligands and the protein

Figure 6 shows the two best conformations (Fig. 6A, B) of the binding poses and interactions between α-glucosidase, hesperidin, and BPA. Figure 6A represents the best model of hesperidin in complex with six different models of bisphenol A while Fig. 6B is the binding pose of the best conformation/models of both ligands and the protein. All the binding conformations consistently revealed that both ligands bind at the same binding pocket on the enzyme with hesperidin having a stronger binding affinity than BPA at the same site, which enhanced its potential of dislodging BPA from its binding pocket on the enzyme. Hesperidin, therefore, has the potential to compete for the same binding site as BPA and may inhibit the binding of BPA on its binding pocket on the α-glucosidase three-dimensional enzyme conformations.

#### Protein-ligands interactions

##### FGF-21-ligands interactions

**Fig. 7:**
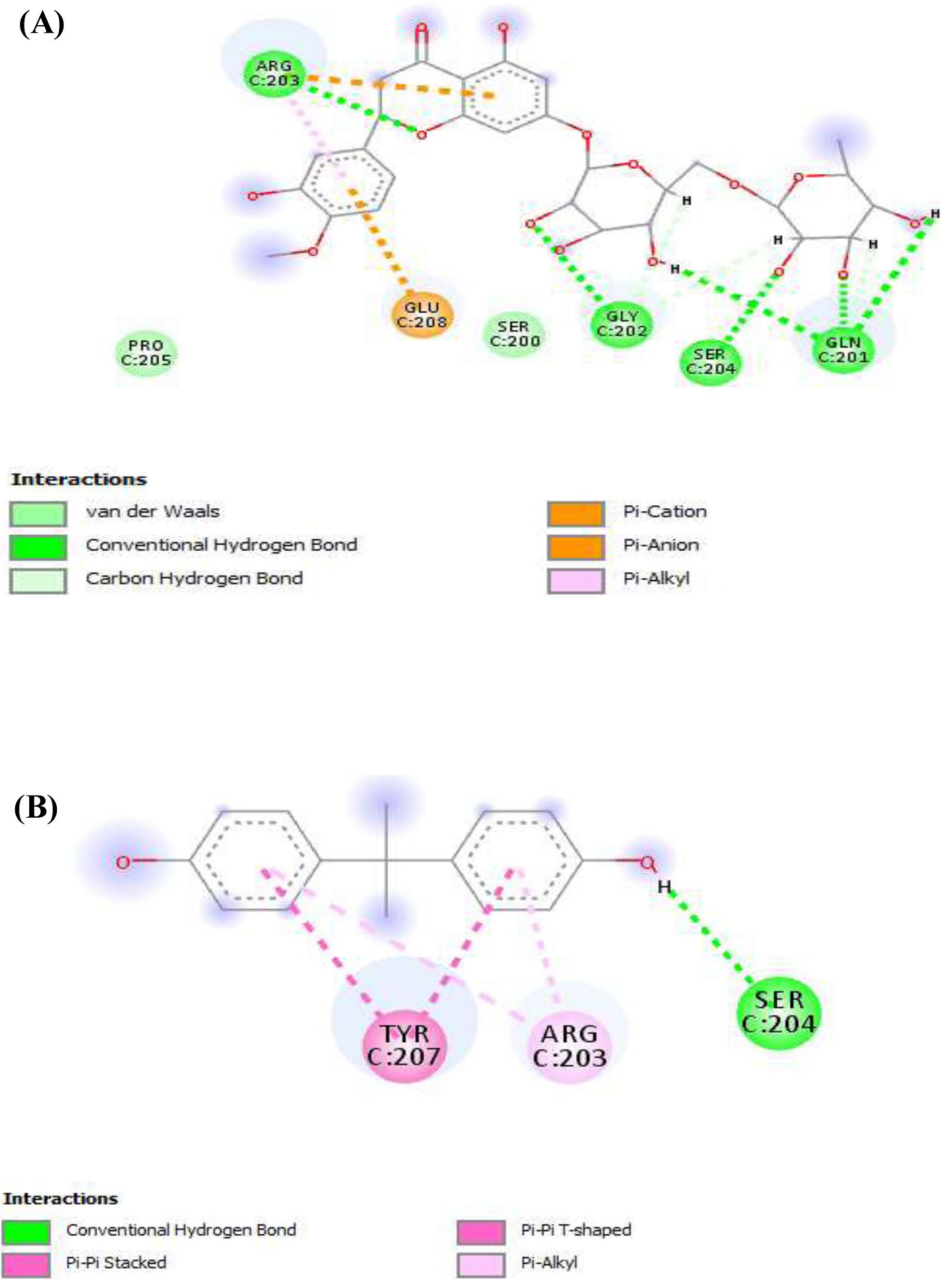
FGF-21-Ligands interaction. **(A)**. Hesperidin. **(B)**. Bisphenol A. Dark green: conventional hydrogen bond; light green: van der Waals; Pink-violet: π-T shape/alkyl; brown: π-cation/anion. Red: unfavorable donor-donor; grey: carbon-hydrogen bond

Figure 7 shows the bidirectional binding interactions of the ligands (Fig. 7A) hesperidin and (Fig. 7B) BPA and the amino acids of the FGF-21 at the binding pocket of the protein. The result revealed that hydrogen and alkyl bonds were the prominent bonds involved in the interactions for both of the ligands, although the alkyl bond was more prominent in the interactions of BPA with the protein. Moreover, hydrogen bond was more prominent for hesperidin-FGF-21 interactions adding to our finding that hesperidin binds stronger than BPA on the protein and perhaps may be an effective inhibitor of BPA. Further molecular inquiry revealed that BPA and hesperidin interacted with the same binding site on the FGF-21 structure through the three main amino acids (Tyr207, Arg203, and Ser204) that made up the binding pocket. Specifically, the ligands interacted with traditional hydrogen bonds in FGF-21, especially using Ser204, which is a strong bond. Besides, along with Ser204, hesperidin has three other amino acids (Gly202, Gln201, and Arg203) with which it also interacted by traditional hydrogen bonding with the binding site.

##### α-amylase ligands interactions

**Fig. 8:**
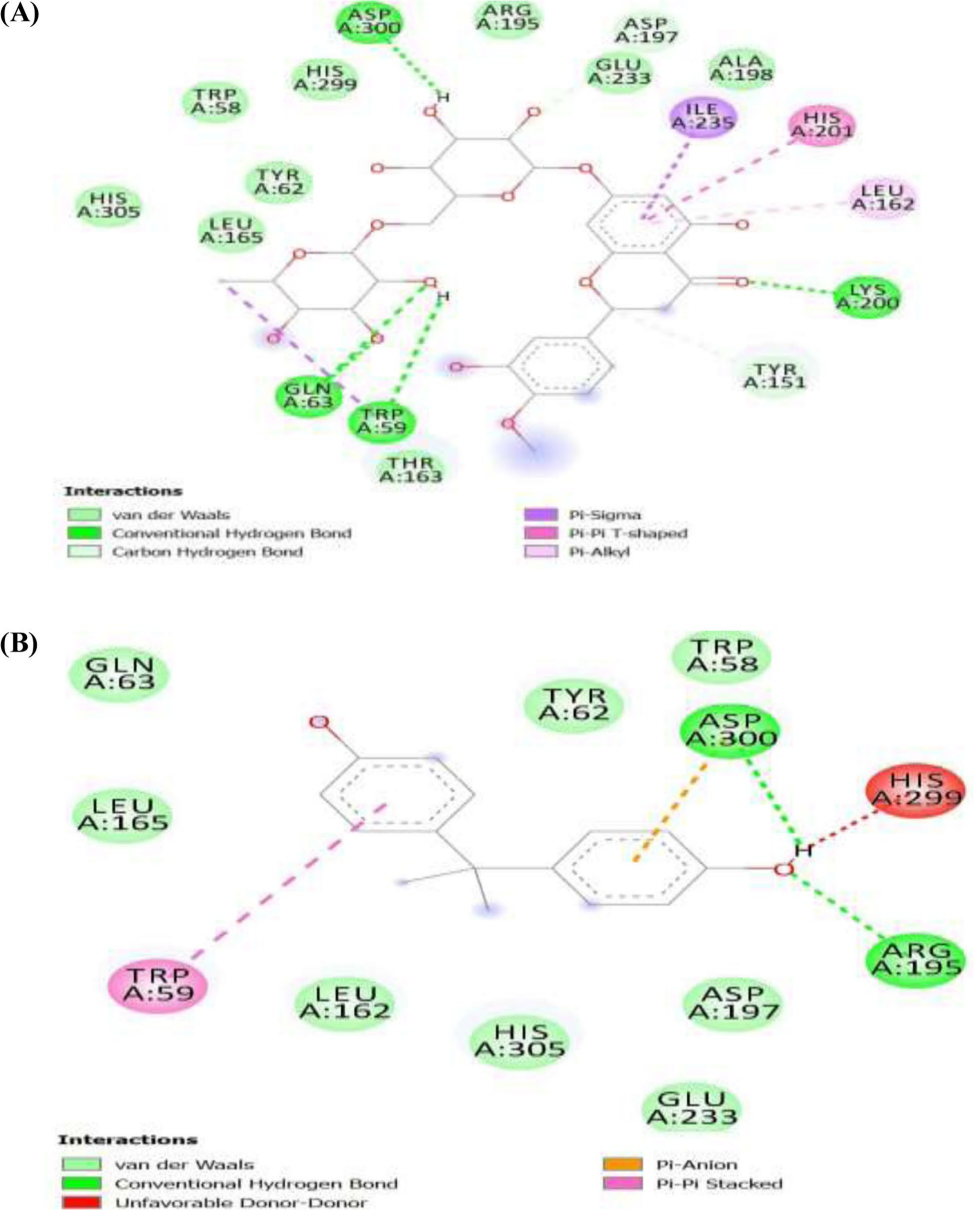
α-amylase-ligands interaction. **(A)**. Hesperidin. **(B)**. Bisphenol A. Deeper green: conventional hydrogen bond; light green: van der Waals; Pink-violet: π-T shape/alkyl/sigma; brown: π-cation/anion. Red: unfavourable donor-donor; grey: carbon-hydrogen bond

Figure 8 shows the bidirectional binding interactions of the ligands (Fig. 8A) hesperidin and (Fig. 8B) BPA and the amino acids of the pancreatic enzyme α-amylase at the binding pocket of the enzyme. The image revealed that hydrogen bond and van der Waals forces were the prominent bonds involved in the interactions for both of the ligands, although van der Waals forces were more prominent in the interactions of BPA with the enzyme. Moreover, hydrogen bond was more prominent for hesperidin-α-amylase interactions adding to our finding that hesperidin binds stronger than BPA on the enzyme and thus would be an effective inhibitor of BPA. The interaction with α-amylase ligands showed that both bisphenol A and hesperidin interacted with traditional hydrogen of various amino acids in the binding pockets of the enzyme as revealed in Fig. 8 (bisphenol A: Asp300, Arg195, hesperidin: Asp300, Lys200, Gln63, and Trp59). Our investigation further revealed that each of the ligands interacted with Asp300.

##### α-glucosidase ligands interactions

**Fig. 9:**
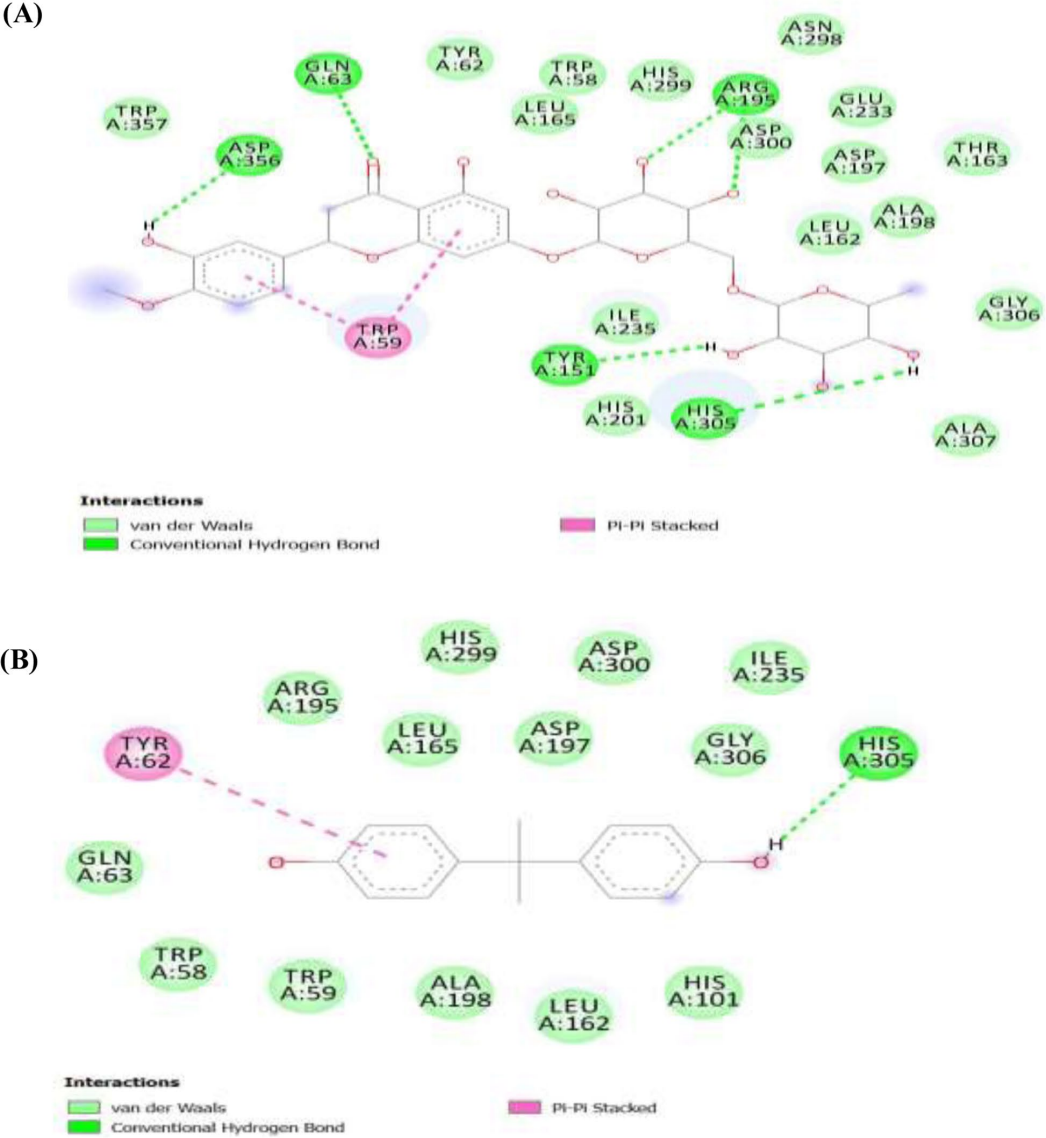
α**-**glucosidase ligands interaction. **(A)**. Hesperidin. **(B)**. Bisphenol A. Dark green: conventional hydrogen bond; light green: van der Waals; Pink: π-π stacked

Figure 9 shows the separate binding interactions of the ligands (Fig. 9A) hesperidin and (Fig. 9B) BPA and the amino acids of the pancreatic enzyme α-glucosidase at the binding pocket of the enzyme. The image revealed that hydrogen bond and van der Waals forces were the major bonds involved in the interactions for both of the ligands, albeit van der Waals forces were more prominent in all. Moreover, hydrogen bonds in hesperidin-α-glucosidase interactions were greater than the hydrogen bonds in BPA-α-glucosidase interactions adding to our finding that hesperidin binds stronger than BPA on the enzyme and thus would be an effective inhibitor of BPA. For the α-glucosidase enzyme, the ligands interacted with the standard hydrogen bond of the enzyme using His305 as shown in Fig. 9. Gln53, Asp356, Arg195, and Try159 also interacted with hesperidin. Although both bisphenol A and hesperidin interacted with the target protein using traditional hydrogen bonds and at least with the main amino acid, hesperidin interacted with more amino acids than BPA indicating the possibility of preventing the binding of BPA and cleaving off bisphenol A from its most potent protein binding sites.

#### ADME/T prediction profile of hesperidin

## Discussion

The human physiological system is frequently exposed to several endocrine disruptor chemicals (EDC) either through environmental toxicants such as heavy metals and/or industrial products such as BPA [37, 38]. These EDCs upon entrance into the body interfere with normal metabolic regulations of the body leading to disease conditions. Bisphenol A is a well-known EDC whose molecular mechanisms of action has remained opaque over the years [39]. Clearer understanding of the mechanistic approach of BPA in causing endocrine disruption and other metabolic dysregulations would be helpful in developing possible new therapeutics against this environmental toxicant. Our study for the first time used in silico molecular techniques to investigate possible novel mechanistic approaches of BPA in inducing endocrine disruption and other metabolic dysregulations. In silico molecular methods is a modern veritable tool in drug discovery and development. Although, modern experimental approaches are able to elucidate ADMET properties with great degree of accuracy, however, they are time-consuming with high cost implications. In silico molecular method therefore remains an efficient key approach in drug discovery and development [40, 41].

In molecular docking, binding free energies or docking scores describe the affinity of a ligand for protein molecules. This is usually denoted with negative values and a higher negative value of docking score results in a higher binding affinity vice versa. Interestingly, our docking score results shown in Table 1, revealed high negative values for all the binding poses between BPA and the three proteins as well as hesperidin and the proteins respectively. Protein-ligand binding interactions is a reversible non-covalent interaction that occur through various molecular mechanics involving conformational changes leading to high affinity and low affinity states. These molecular interactions are essential to all life processes and thus are strongly considered in drug discovery and development of new therapeutics [42]. Our results revealed that BPA can bind with FGF-21, α-amylase, and α-glucosidase respectively, suggesting a novel mechanistic approach of BPA interference with glucose metabolism through interactions with these metabolic regulators. FGF-21, α-amylase, and α-glucosidase are key proteins involved in the metabolism of glucose in the body [43–45]. Although inhibition of α-amylase and α-glucosidase may be seen to be a potential action of BPA in regulating glucose level, it is not clear how this may manifest in the physiological system in vivo studies. Inhibiting α-amylase and α-glucosidase in addition to other metabolic targets of BPA may rather exacerbate glucotoxicity and metabolic dysfunctions in the body. FGF-21 is an indicator of metabolic syndrome and endocrine dysfunction [46] and BPA binding affinity to FGF-21 may be a novel mechanism of action of BPA in disrupting metabolic regulations leading to metabolic derangement in the body.

**Table 1:** Molecular docking scores for the best pose of the ligands

Protein	Ligand	Binding energy (Kcal/mol)
FGF21	Hesperidin	− 5.80
	Bisphenol A	− 4.40
α-amylase	Hesperidin	− 9.60
	Bisphenol A	− 7.20
α-glucosidase	Hesperidin	− 9.60
	Bisphenol A	− 7.10

Interestingly, in Figs. 4, 5, and 6, we further demonstrated that hesperidin binding to FGF-21, α-amylase, and α-glucosidase interfered with the binding of BPA to these metabolic regulators. This is further evidenced in the higher binding affinity/negative values of hesperidin in all the binding poses with the proteins investigated. Hesperidin is an emerging natural product recently revealed to have strong therapeutic potentials for the treatment of metabolic diseases such as diabetes and obesity [30]. Our results revealed that although bisphenol A bound with all 8, 6, or 7 of its conformational models to specific sites on the proteins; hesperidin bound at the same site with BPA with stronger affinity demonstrating that hesperidin outcompeted with BPA on its binding pockets on FGF-21, alpha-amylase, and α-glucosidase 3-D structure. Proteins usually recruit its residues during ligands binding, which subsequently enhance its interactions with the ligands especially at the binding pockets. The greater the residues involved in the binding, the greater the affinity of the protein to the ligand [47, 48]. In all the three proteins investigated, the binding of hesperidin led to the recruitment of more residues of the proteins than when BPA bound. Thus further indicating that hesperidin has greater binding affinity than BPA. Hesperidin may therefore be a promising strong inhibitor of BPA binding especially against these proteins investigated. The interactions of the ligands (BPA and hesperidin) with the proteins as competitive inhibitors are shown in Figs. 7, 8, and 9.

To further position hesperidin as a strong lead compound in drug development, we conducted ADME/T studies to enhance our understanding of its druggability. In decision-making to boost the success rates in the early drug development process, knowledge relating to the drug-like appearance of compounds is imperative [34]. Interestingly, many methods and instruments can be used in testing a molecule’s physicochemical properties that can influence its pharmacokinetic and pharmacodynamic properties in vivo [49]. ADME properties of hesperidin were evaluated and the selected properties correlated with metabolism, cell permeation, and bioavailability. Hesperidin proved to be a strong inhibitor of bisphenol A binding, however violated Lipinski’s five-molecular weight rule of 610.56, H-bond Acceptor 15, and H-bond Donor 8 (Table 2). Moreover, investigation of human intestinal absorption (HIA) usually reveals human intestinal permeability as shown in Table 3 and better absorption through the intestine is reflected by a probability value closer to 1 [34]. Interestingly, hesperidin displayed a strong absorption value of 0.8161 and displayed near values for the in silico simulation (Caco-2) in the human cell line used. As shown from the Ames test, hesperidin would be neither mutagenic nor carcinogenic in vivo. In addition, the LD_50_ of hesperidin was high, which indicates that hesperidin would not be toxic in vivo even at a high dosage.

**Table 2:** Lipinski rule of five from SwissAdme server

Physical property	Value
Molecular weight	610.56^*^
LogP	2.6
H-bond acceptor	15^*^
H-bond donor	8^*^
Rotatable bonds	7

^*^Lipinski rule of five violations

**Table 3:** In silico absorption and toxicity profile of the compounds as obtained from the AdmetSar server.

Absorption/toxicity profile	Value	Probability
Human blood-brain barrier (BBB)	−	0.9570
Intestinal absorption (HIA)	+	0.8161
Caco-2	−	0.8816
Plasma protein binding	1.096436381	1.00
AMES Test	−	0.6600
Carcinogenicity	−	0.9714
UGT catalysed	+	0.6000
Acute oral toxicity (LD50; kg/mol)	2.257	
Hepatotoxicity	+	0.6500

In silico distribution profile showed that hesperidin is a non-substrate and non-inhibitor of P-glycoprotein (P-GP) (Table 4). P-glycoprotein is one of the major drug transporters that is involved in the uptake and efflux of drugs and xenobiotics in and out of the cell. This process significantly affects plasma and tissue concentrations of such drug in addition to their therapeutic profile [50]. P-glycoprotein is strongly implicated in drug-drug interactions in the physiological system and understanding its interactions with drugs are necessary in new drug development [51]. It has been shown that drugs that induce P-glycoprotein, has the ability to reduce the bioavailability of some other drugs. Additionally, inhibitors of P-glycoprotein, could increase the bioavailability of susceptible drugs transported by P-glycoprotein [52]. Interestingly, our investigation revealed that hesperidin is a non-substrate and non-inhibitor of P-glycoprotein, thus a strong pharmacological advantage to hesperidin as there may not be significant alterations in its therapeutic effects due to the activity of P-glycoprotein when they are administered into the body.

**Table 4:** In silico distribution profile of hesperidin as obtained from the AdmetSar server

Distribution substrate/inhibitor	Profile
p-gp substrate/inhibitor probability	Non-substrate/non-inhibitor
CYP-2C9 substrate/inhibitor	Non-substrate/non-inhibitor
CYP-2D6 substrate/inhibitor	Non-substrate/non-inhibitor
CYP-3A4 substrate/inhibitor	Substrate/non-inhibitor
CYP-1A2 inhibitor	Non-inhibitor
CYP-2C19 inhibitor	Non-inhibitor
CYP inhibitory promiscuity	0.6670

Cytochrome P450 is a family of microsomal enzymes involved in the metabolism of xenobiotics including drugs and many drug-drug interactions are as a result of alterations in the metabolism of CYP450 [36, 53]. They however can be inhibited or induced by drugs and their metabolites, leading to drug-drug interactions with significant clinical relevance [54]. Such drug-drug interactions could lead to adverse effects or therapeutic failures of clinically administered drugs [55]. They are therefore strongly considered in the development of new drug candidates especially as it relates to drug-drug interactions [56, 57]. We therefore assessed the cytochrome P450 inhibition profile for hesperidin for the five major isoforms (2C9, 2D6, 3A4, 1A2, and 2C19), which are involved in the metabolism of many drugs. Our result revealed that hesperidin is neither a substrate nor an inhibitor of these isoforms (2C9 and 2D6) of cytochrome P450. It was also specifically found not to be an inhibitor of 3A4, 1A2, and 2C19 isoforms although a substrate 3A4 cytochrome P450 isoform. Inhibitors of CYP450 enzymes usually block the metabolic activity of one or more CYP450 enzymes [55]. Moreover, the extent to which an inhibitor affects the CYP450 metabolism of a specific drug depends on many factors such as drug dosage and the inhibitor’s ability to effectively bind to the enzyme. Interestingly, our result revealed that hesperidin do no inhibit any of the major isoforms of CYP450, which is a strong feature of good drug candidate.

More so, several drugs interact with the CYP450 system in many ways. Some drugs may be metabolized by only one CYP450 enzyme whereas others may be metabolized by more than one isoforms of the enzyme. Drugs that are substrates and/or inhibitors of any of the cytochrome P450 isoforms are known to interfere with the metabolism of others drugs that depend on the isoform leading to drug-drug interactions [58]. However, the more CYP450 enzymes they are substrate to, the more they are able to cause drug-drug interactions [59]. Interestingly, our study revealed that hesperidin is a substrate of only CYP3A4 isoform of CYP450 enzyme system, thus making it to have lower risk of triggering drug-drug interactions when it is administered into the body for therapeutic purposes (Table 4).

In general, hesperidin is an active inhibitor of bisphenol A binding to FGF-21, alpha-amylase, and alpha-glucosidase, which are known regulators of metabolism. Conversely, despite the revealed potentials of hesperidin to inhibiting these target proteins, its high molecular weight could lead to decreased solubility and bioavailability within the physiological system when administered for therapeutic purposes. However, this can be improved through pharmaceutical modifications and optimizations of the hesperidin molecular structure to produce derivatives with greater solubility and bioavailability in vivo, thus enhancing it prospects of meeting up with Lipinski’s five-molecular weight rule.

## Conclusions

In this present study, we showed perhaps a novel mechanistic approach of BPA endocrine disruption through inhibition of FGF-21, α-amylase, and α-glucosidase, which are key endocrine regulators of glucose metabolism. Interestingly, hesperidin a natural product strongly disrupted the binding of BPA to these metabolic regulators by interfering with its binding at the same binding pocket of the regulators with stronger affinity with α-amylase, and α-glucosidase producing the highest binding affinity with hesperidin. Hesperidin further showed a promising ADME/T profile hence enhancing its potential for further drug development. Hesperidin may be a promising drug candidate for the development of therapeutics against metabolic-related diseases.

## Data Availability

We have included all the data.

## Abbreviations

FGF-21: Fibroblast Growth Factor-21
BPA: Bisphenol A
ADME/T: Administration, Distribution, Metabolism, Excretion, and Toxicity
EDC: Endocrine disrupting chemical
PDB: Protein Data Bank
CYP: Cytochrome
